# *Rhapontici Radix* Extract Inhibits Colorectal Intraepithelial Neoplasia by Regulating the YAP/PI3K-AKT Signaling Pathway: Evidence from Animal Models, Organoids, and Cytological Studies

**DOI:** 10.3390/biomedicines14050956

**Published:** 2026-04-22

**Authors:** Fan Xiao, Zhilu Lei, Bo Wu, Zhenyu Niu, Guifang Deng, Linjing Su, Yaqian Cao, Kerong Qi, Xiaoqing Sun, Qike Tan, Junyu Ke, Yanwu Li

**Affiliations:** 1 Science and Technology Innovation Center, Guangzhou University of Traditional Chinese Medicine, Guangzhou 510405, China; m15512221695@163.com (F.X.); jaderay826@163.com (Z.L.); 20201110935@stu.gzucm.edu.cn (B.W.); 20211110932@stu.gzucm.edu.cn (Z.N.); dgf1834362898@163.com (G.D.); 13787353334@163.com (L.S.); zydrcyq@163.com (Y.C.); 13609277532@163.com (K.Q.); sunxiaoqing0312@163.com (X.S.); 13246142472@163.com (Q.T.); 2 School of Basic Medical Sciences, Guangzhou University of Traditional Chinese Medicine, Gaozhou 525200, China

**Keywords:** colorectal epithelial neoplasia (CR-EN), *Rhapontici Radix*, YAP and PI3K/AKT signaling pathway, organoid models

## Abstract

**Background:** Colorectal intraepithelial neoplasia (CR-EN) is a precursor lesion of colitis-associated colorectal cancer (CAC). This study investigated the interventional effects and molecular mechanisms of Rhapontici Radix extract on CR-EN. **Methods:** An azoxymethane/dextran sulfate sodium (AOM/DSS)-induced mouse model of colonic intraepithelial neoplasia, bioinformatics analysis, organoid models, and HCT116 cell experiments were employed, coupled with histopathological examination, inflammatory cytokine detection, Western blot, immunofluorescence, and HPLC-MS/MS. **Results:** The results showed that the YAP/AKT-PI3K signaling pathway is aberrantly activated in CRC. Rhapontici Radix extract ameliorated colonic pathology, suppressed inflammatory responses, and remodeled gut microbiota composition in model mice. The extract selectively inhibited the proliferation of CR-EN organoids by downregulating Ki67 and β-catenin while upregulating p53, and suppressed the proliferation, colony formation, and migration of HCT116 cells. Mechanistically, the extract modulated the YAP/PI3K/AKT pathway by upregulating phosphorylated YAP (p-YAP) and downregulating phosphorylated AKT (p-AKT), phosphorylated PI3K (p-PI3K), and their downstream targets p-SRC and c-MYC. **Conclusions:** This study suggests that Rhapontici Radix extract intervenes in inflammation-associated carcinogenesis through a multi-pathway, multi-target strategy, offering potential therapeutic targets for CAC prevention and treatment.

## 1. Introduction

Colitis-associated colorectal cancer (CAC) is one of the most prevalent malignant tumors worldwide, characterized by high metastatic potential, frequent recurrence, and poor prognosis, thus representing a severe threat to human health [1,2]. Colorectal epithelial neoplasia (CR-EN), a key precancerous lesion in the pathogenesis of CAC, is of great clinical significance for elucidating the underlying etiology of CAC. It is widely acknowledged that the pathological progression of CAC follows a canonical sequence: “inflammation—epithelial neoplasia—carcinogenesis” [3,4]. In recent years, the rise in CR-EN cases has also contributed to the increase in CAC incidence [5,6]. Therefore, exploring effective therapeutic interventions at the CR-EN stage may offer novel strategies for delaying colorectal tumorigenesis and facilitating early cancer prevention [7].

*Rhapontici Radix* (RR) is traditionally used in Traditional Chinese Medicine for the stomach and large intestine meridians. It is employed to clear heat, detoxify, and treat abscesses and pus. The “Shennong Bencao Jing” records that it is “bitter, salty, and cold, mainly treating skin heat, ulcers, carbuncles, dampness, and promoting lactation.” It is often used in clinics to treat ulcerative colitis and colon polyps, frequently in combination with other herbs to achieve significant efficacy [8]. Modern pharmacological studies have demonstrated that multiple active constituents in Rhapontici Radix, including saponins, flavonoids, and polysaccharides, exert significant anti-tumor activities in various malignant tumor models, such as gastric cancer, breast cancer, and oral cancer. However, its exact role and underlying molecular mechanisms in the “inflammation–carcinogenesis” cascade of CAC remain poorly understood [9,10,11].

Our previous studies have shown that a heat-clearing, blood-stasis-resolving, and spleen-strengthening formula with Rhapontici Radix as the principal herb suppresses the pathological progression of colon cancer in CAC mouse models. On this basis, the present study further investigates the inhibitory effects of Rhapontici Radix on colorectal epithelial neoplasia during CAC development and its potential molecular mechanisms [12].

## 2. Materials and Methods

### 2.1. Bioinformatics Analysis

The expression levels of Yes-associated protein (YAP), protein kinase B (AKT), and phosphoinositide 3-kinase (PI3K) family genes (*YAP1*, *AKT1*, *AKT2*, *PIK3R2*, *PIK3R3*) in Colon Adenocarcinoma (COAD) were analyzed using data from the Genotype-Tissue Expression Project (GTEx) and The Cancer Genome Atlas (TCGA) databases. Raw data from colorectal cancer tissues and adjacent normal colorectal tissues were downloaded, normalized, and processed to compare the expression differences in target genes between the two groups.

### 2.2. Preparation of Freeze-Dried Powder

*Rhapontici Radix* was acquired from Suzhou Tianling Traditional Chinese Medicine Co., Ltd. (Batch No.: 230920010, Suzhou, China) and was first soaked in distilled water at a weight-to-volume ratio of 1:10 for 2 h before undergoing three consecutive extractions via the traditional decoction method with each extraction lasting 1 h. The obtained filtrates were combined and concentrated under reduced pressure to approximately 50 mL and this concentrated solution was then frozen at −80 °C and subjected to freeze-drying. The resulting freeze-dried powder was sealed in an airtight container and stored at −20 °C for subsequent experimental use.

### 2.3. Ultra-High Performance Liquid Chromatography Mass Spectrometry Analysis

Powdered *Rhapontici Radix Freeze-Dried Powder* (≈0.2 g) was accurately weighed, extracted with 5 mL 70% methanol by ultrasonication for 30 min, centrifuged at 12,000 r/min and 4°C for 10 min. The supernatant was filtered through a 0.22 μm microporous membrane, and the filtrate was used as the test solution.

An Agilent 1290 UHPLC system with an Agilent ZORBAX Eclipse Plus C18 column (2.1 × 100 mm, 1.8 μm, Santa Clara, CA, USA) was used. Column temperature: 35°C; flow rate: 0.2 mL/min; injection volume: 2 μL. Mobile phase A: 0.1% formic acid-water, B: acetonitrile. Gradient elution: 0–0.5 min (95% A); 0.5–18 min (95% A→40% A); 18–21 min (40% A→5% A); 21–21.1 min (5% A→95% A) for equilibration.

An Agilent 6545XT Q-TOF mass spectrometer (Santa Clara, CA, USA) with ESI source was used, and data were collected in both positive and negative ion modes. Key parameters: capillary voltage 4000 V, collision voltage 120 V, fragmentor voltage 65 V, nozzle voltage 500 V; ion source temperature 350°C, sheath gas flow 12 L/min, nebulizer pressure 45 psi; scan range *m*/*z* 50–1700, collision energy (30 ± 15) eV.

### 2.4. Animals and Ethics

Six- to eight-week-old male C57BL/6 mice were purchased from the Guangdong Experimental Animal Center (License No.: SYXK9(Yue)2023-0347, Guangzhou, China) and were housed under specific pathogen-free (SPF) conditions with continuous access to food and water as well as appropriate bedding and nesting materials to ensure their comfort. The temperature in the breeding environment is maintained between 22 °C and 26 °C, with humidity kept at 50% to 70%, and management is conducted using a day-night lighting interruption system. All experimental procedures involving the mice were performed in strict compliance with the regulations formulated by the Animal Ethics Committee of Guangzhou University of Chinese Medicine (Approval No.: 20240305041).

### 2.5. Establishment of Colorectal Epithelial Neoplasia Mouse Model

A murine model of colorectal epithelial neoplasia was constructed in C57BL/6 mice through a combined induction protocol with AOM (azoxymethane; Sigma-Aldrich, St. Louis, MO, USA, Catalog No.: 25843-45-2) and DSS (dextran sulfate sodium; Sigma-Aldrich, Catalog No.: 160110) [13,14,15]. After a 3-day acclimation period, all animals except those in the blank control group were given a single intraperitoneal injection of AOM at a dose of 12 mg/kg body weight which was prepared as a 1 mg/mL solution in physiological saline. Following this injection, the mice were fed a regular diet for 7 days and were subsequently provided with distilled water supplemented with 1.5% DSS for 7 days which was then replaced with standard drinking water for a 14-day recovery phase. This sequence constituted one modeling cycle with a total duration of 3 weeks and the cycle was repeated twice more thereby resulting in three cycles over a 9-week period. During the entire experimental course, daily monitoring was performed to record the mice’s body weight, activity levels, and fecal characteristics and when a mouse showed a weight loss ranging from 10% to 20% of its initial weight it received either 1 mL of physiological saline via intraperitoneal injection or softened feed to alleviate dehydration. Euthanasia was performed when the mouse body weight decreased by ≥20% or severe disease symptoms appeared. The blank control group was consistently provided with regular drinking water throughout the entire experiment.

### 2.6. Experimental Grouping and Administration

A total of 60 SPF-grade 10-week-old male C57BL/6 mice, which were randomly divided into five groups (*n* = 12 per group) based on their body weight including the blank control group (Control), the model group (Model), the positive drug control group (Mesalazine administered at 150 mg/kg/day), the low-dose *Rhapontici Radix* group (*Rhapontici Radix*-L corresponding to 1.3 g of crude herb per kg per day), and the high-dose *Rhapontici Radix* group (*Rhapontici Radix*-H equivalent to 2.6 g of crude herb per kg per day). Treatment administration began on day 49 after the completion of the second DSS cycle and both the freeze-dried powder of *Rhapontici Radix* and mesalazine were dissolved in distilled water and administered via gavage while the blank control and model groups received the same volume of distilled water through the same gavage method. All treatment regimens were maintained until the end of the experiment on day 70.

### 2.7. Organizational Pathology Analysis

Paraffin sections of mouse colon tissue were stained with hematoxylin and eosin (H&E) for pathological analysis.

### 2.8. ELISA Detection

Levels of TNF-α (Brand: Shanghai Jianglai Biological Technology Co., Ltd., Shanghai, China, Catalog No.: JLW10484), IL-10 (Brand: Shanghai Jianglai Biological Technology Co., Ltd., Catalog No.: JLW20242), IL-6 (Brand: Shanghai Jianglai Biological Technology Co., Ltd., Catalog No.: JLW20268), and IL-1β (Brand: Shanghai Jianglai Biological Technology Co., Ltd., Catalog No.: JLW18442) in colon tissue samples from each group were measured using ELISA.

### 2.9. Western Blot

The colon tissue of the mouse was cracked on the ice with a RIPA buffer with inhibitors, and the supernatant was collected after centrifugation. The protein concentration was determined by BCA method, and an equal amount of protein samples were denatured and transferred to the polyfluoroethylene (PVDF) membrane after SDS-PAGE electrophoresis. After the PVDF membrane was sealed with 5% skimmed milk powder, it was incubated with the first antibody at 4 °C for overnight, and then incubated with the second antibody with horseradish peroxidase (HRP) malated at room temperature for 1 h. After enhanced chemiluminescence (ECL) detection, the image was captured and quantitatively analyzed with ImageJ 1.54g software, and β-actin was used as the internal reference control.

The primary antibodies employed were: AKT (Brand: CST, Danvers, MA, USA, Catalog No.: 4691S, diluted 1:1000); p-AKT (Brand: CST, Catalog No.: 4060S, diluted 1:1000); PI3K (Brand: CST, Catalog No.: 4257S, diluted 1:1000); p-PI3K (Brand: CST, Catalog No.: 4228S, diluted 1:1000); YAP1 (Brand: Wanlei Biotechnology, Liaoning, China, Catalog No.: WL03624,diluted 1:1000); p-YAP1 (Brand: Wanlei Biotechnology, Catalog No.: WL05079, diluted 1:1000); β-actin (Brand: Proteintech Group Inc., Rosemont, IL, USA, Catalog No.: 10230-1-AP, diluted 1:5000); p53 (Brand: Proteintech Group Inc., Catalog No.: 10442-1-AP, diluted 1:1000); β-catenin (Brand: HUABIO, Woburn, MA, USA, Catalog No.:ET1601-5, diluted 1:1000);KI67 (Brand: HUABIO, Catalog No.:HA721115, diluted 1:1000).

### 2.10. Immunofluorescence Staining

Tissue sections were deparaffinized, rehydrated, and subjected to antigen retrieval in citrate buffer. After blocking with 5% BSA for 30 min at room temperature, sections were incubated with primary antibodies overnight at 4°C. Then, fluorescent secondary antibodies were added and incubated for 1 h at room temperature in the dark. DAPI was used to stain cell nuclei. Images were captured under a fluorescence microscope.

The primary antibodies employed were: p-YAP1 (Brand: Wanlei Biotechnology, Catalog No.: WL05079, diluted 1:500); p-SRC (Brand: CST, Catalog No.: cat.#4943, diluted 1:40); c-Myc (Brand: Wanlei Biotechnology, Catalog No.:WL01781, diluted 1:200).

### 2.11. Immunofluorescence Staining for Organoids

Organoids were fixed with 4% paraformaldehyde, permeabilized with 0.3% Triton X-100, and blocked with 5% BSA for 30 min at room temperature. Then, they were incubated with primary antibodies overnight at 4 °C, followed by fluorescent secondary antibodies for 1 h at room temperature in the dark. Nuclei were stained with DAPI. Images were acquired using a fluorescence microscope.

The primary antibodies employed were: p-AKT (Brand: CST, Catalog No.: 4060S, diluted 1:400); p-PI3K (Brand: CST, Catalog No.: 4228S, diluted 1:400); YAP1 (Brand: Wanlei Biotechnology, Catalog No.: WL03624,diluted 1:400); p53 (Brand: Proteintech Group Inc., Catalog No.: 10442-1-AP, diluted 1:200); β-catenin (Brand: HUABIO, Catalog No.:ET1601-5, diluted 1:200); KI67 (Brand: HUABIO, Catalog No.:HA721115, diluted 1:1000).

### 2.12. Extraction and Culture of Colonic Organoids

Colon specimens obtained from normal mice and those with epithelial neoplasia were thoroughly washed and then chopped before being incubated in an enzymatic digestion solution for 30 min and the digestion process was subsequently terminated. The resulting mixture was filtered through a 100 μm cell filter and centrifuged at 300 g to collect the cells which were then washed three times with a designated basic culture medium and encapsulated in an organoid matrix gel to support three-dimensional culture. The prepared mixture was transferred to a 24-well plate and allowed to solidify in an incubator for 20 min before 500 μL of culture medium was added. Colonic Organoids were cultured statically in an incubator at 37 °C with 5% CO_2_, and passaged every 5–7 days. The experimental reagents utilized included colorectal cancer organoid kits (Cacm), organoid culture extracellular matrix (ECM), mouse colon organoid kits (Mcm), organoid matrix gel, tumor tissue digestion solution, organoid dissociation solution, anti-adhesion wash solution, and red blood cell lysis solution all sourced from Bozhen Biotechnology (Suzhou, China) Co., Ltd. with catalog numbers K2103-CR, M315066, K2204-MC, K601003, E238001, E238002, and E238010 respectively.

### 2.13. Organoid Grouping and Administration

Four groups of colonic epithelial neoplasia organoids were established, including one model group and three *Rhapontici Radix* extract treatment groups. The model group consisted of AOM/DSS-induced colonic epithelial neoplasia organoids without pharmacological intervention and served as the control benchmark while the treatment groups were administered *Rhapontici Radix* extract at concentrations of 1000 μg/mL, 500 μg/mL, and 250 μg/mL respectively. The corresponding lyophilized powder solution was added to the tumor organoid culture system, and the subsequent effects of the drug on the organoids were evaluated using specific detection and analytical methods.

### 2.14. ATP Detection

Cultured plates were removed from the incubator and allowed to equilibrate to room temperature for 10 min before the detection reagent (Bozhen Biotechnology (Suzhou, China) Co., Ltd., Catalog No.: E238003) which had also been equilibrated to room temperature was added to the plates in a 1:1 volume ratio. The plates were incubated at room temperature for an additional 20 min and the chemiluminescent signal was then measured at a wavelength of 560 nm.

### 2.15. MTT Assay

By redissolving the freeze-dried *Rhapontici Radix* extract in the modified Dulbecco’s Eagle culture medium (DMEM), the concentration is 1 g/mL, and then sterilized by a 0.45 μm syringe filter, and then diluted to the working concentration of 100 Mg/mL. The cells are inoculated in a 96-hole plate, treated after 24 h of wall culture, and MTT solution (Sigma, catalog number: M2128) is added 24, 48 and 72 h after treatment, and then incubated for 2 h. Carefully absorb the culture medium, add 150 μL dimethyl sulfur (DMSO) to each hole, and mix gently for 10 min, and measure and quantify the absorbance at a wavelength of 570 nm.

### 2.16. Wound Healing Assay

HCT116 cells were cultured in 6-well plates, and once the cells reached confluence, a vertical scratch was created on the cell monolayer using a 200 μL pipette tip. The wells were then rinsed with phosphate-buffered saline (PBS) to remove non-adherent cells, and after the designated treatment period, ImageJ software was utilized to quantify the cell migration distance at 12-h and 24-h time points.

### 2.17. Cell Cloning Assay

HCT116 cells in the logarithmic growth phase were harvested, trypsinized to form a single-cell suspension, and seeded into 6-well plates. After 24 h of incubation, the cells were treated with Rhaponticum uniflorum extract at gradient concentrations. Subsequently, the medium was replaced with drug-free complete medium, and the cells were statically cultured in an incubator for 7–14 days. The medium was refreshed every 3–4 days during the culture period until macroscopically visible cell clones were formed.

Following incubation, the medium was aspirated, and the cells were rinsed twice with phosphate-buffered saline (PBS). The cells were then fixed with 4% paraformaldehyde for 15–30 min. Residual fixative was removed by gentle rinsing with running water, and the cells were air-dried naturally. Images were acquired under a light microscope, and the number of cell clones was counted for statistical analysis.

### 2.18. Statistical Analysis

Prior to analyzing intergroup differences, the normality of data distribution was first examined using GraphPad Prism software (Version 9.0, GraphPad Software, San Diego, CA, USA). Data are presented as mean ± standard deviation (SD) from at least three independent experiments. Statistical significance was determined using one-way analysis of variance (ANOVA) followed by Tukey’s post hoc test for multiple comparisons. For comparisons between two groups, a two-tailed unpaired *t*-test was used. A value of *p* < 0.05 was considered statistically significant.

## 3. Results

### 3.1. The Expression of YAP, AKT, and PI3K in Colorectal Cancer

**Figure 1:**
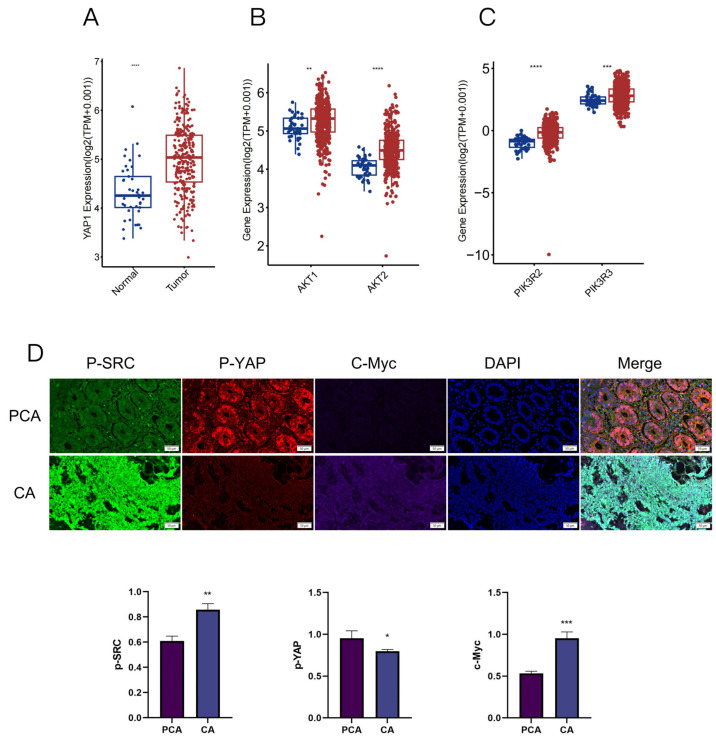
Expression of YAP, AKT, and PI3K in Colorectal Cancer. (**A**–**C**) The expression profiles of YAP, AKT, and PI3K in colorectal cancer were analyzed using the CTEX and TCGA databases. (**D**) Immunofluorescence (IF) staining demonstrates the protein expression levels of p-SRC, p-YAP, and c-Myc in tissue samples in comparison with normal colorectal (PCA) tissues. Statistical significance is indicated as * *p* < 0.05, ** *p* < 0.01, *** *p* < 0.001, and **** *p* < 0.0001 when compared to the model group.

A comprehensive analysis of the expression levels of Yes-associated protein (YAP), protein kinase B (AKT), and phosphoinositide 3-kinase (PI3K) in colorectal cancer was conducted by utilizing data from the GTEx and TCGA databases (Figure 1A–C). Our bioinformatics assessment revealed a significant elevation in the mRNA expression levels of YAP1, AKT1, AKT2, PIK3R2 and PIK3R3 in COAD tissues when compared with normal colorectal tissues. Immunofluorescence assays further demonstrated that the expression levels of phosphorylated SRC (p-SRC) and c-Myc were notably higher in cancerous tissues than in adjacent non-cancerous tissues, while conversely, the expression level of phosphorylated YAP (p-YAP) was significantly reduced in cancer tissues relative to their adjacent non-cancerous counterparts (Figure 1D). These findings indicate that YAP, AKT, and PI3K are overexpressed in colorectal cancer and underscore their potential involvement in the initiation and progression of this malignant tumor.

### 3.2. Chemical Composition Analysis of Rhapontici Radix Extract

**Figure 2:**
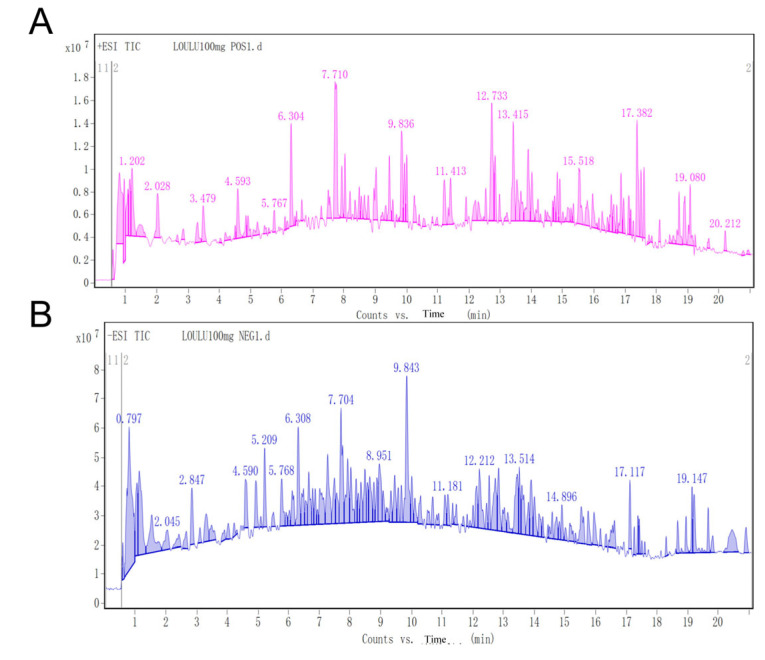
Analysis of the ultra-high-performance liquid chromatography-mass spectrometry of the extract of Rhapontici Radix. (**A**) Negative ion flow chromatogram. (**B**) Positive ion flow chromatogram.

We identified the chemical components in the extract of Rhapontici Radix using HPLC-MS/MS. By looking at the chromatograms for positive ions (Figure 2A) and negative ions (Figure 2B), along with data on secondary fragment ions and molecular weights, we identified a total of 59 different compounds in positive ions and 94 in negative ions (Supplemental Materials) including alkaloids, terpenoids, and flavonoids.

### 3.3. Effects of Rhapontici Radix Extract on AOM/DSS-Induced Colorectal Epithelial Neoplasia Mouse Model

**Figure 3:**
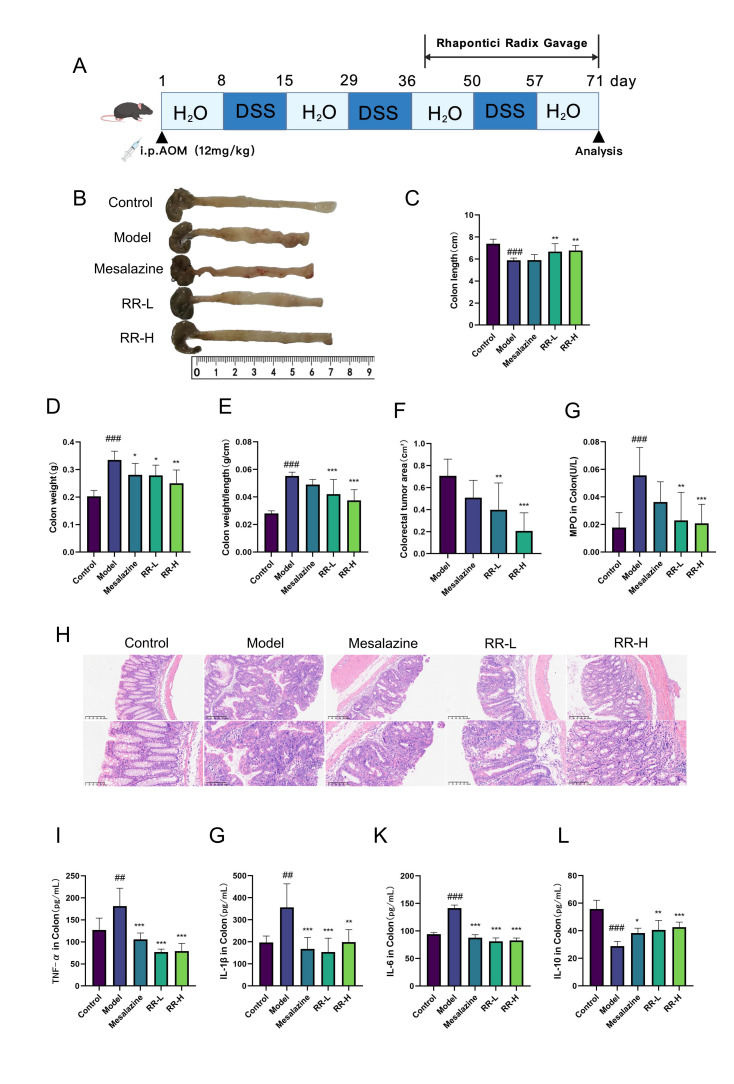
The impact of *Rhapontici Radix* extract on a mouse model exhibiting colonic intraepithelial neoplasia induced by AOM/DSS was investigated. (**A**) A schematic representation detailing the administration protocol for mice diagnosed with colonic intraepithelial neoplasia is presented. Created with BioGDP.com. (**B**) The colons of mice within various treatment cohorts exhibiting colonic intraepithelial neoplasia were analyzed. (**C**–**F**) A comparative assessment of colon length, weight, specific weight, and tumor area was conducted across different treatment groups of mice with colonic intraepithelial neoplasia. (**G**) Myeloperoxidase (MPO) levels in the colons of colonic intraepithelial neoplasia mice subjected to various treatments were evaluated. (**H**) Hematoxylin and eosin (HE) staining results of the colon from colonic intraepithelial neoplasia mice across different treatment conditions are illustrated. (**I**–**L**) A comparative analysis of pro-inflammatory cytokines, including TNF-α, IL-6, IL-1β, and IL-10, was performed in the colons of colonic intraepithelial neoplasia mice across varied treatment groups. Statistical significance was determined with * *p* < 0.05, ** *p* < 0.01, and *** *p* < 0.001 when compared to the model group. Statistical significance was determined with ## *p* < 0.01 and ### *p* < 0.001 when compared to the control group.

An AOM/DSS-induced colorectal epithelial neoplasia mouse model was established in this investigation and varying dosages of *Rhapontici Radix* extract were administered to evaluate its potential efficacy in alleviating colorectal epithelial neoplasia in the experimental mice (Figure 3A). The outcomes indicated a significant reduction in colon length among model mice and the emergence of polyp-like lesions within the intestinal wall while in comparison to the model cohort the *Rhapontici Radix* extract-treated group exhibited an increase in colon length a decrease in colon weight and a reduction in tumor area thereby suggesting that *Rhapontici Radix* extract possesses a pronounced capacity to improve colon health and inhibit tumor development within the colonic mucosa of model mice (Figure 3B–F).

Histopathological analysis via HE staining illustrated substantial glandular architectural distortion a notable decrease in goblet cell populations loss of nuclear polarity in epithelial cells and varying degrees of dysplasia and adenomatous transformation in the colonic mucosa of the model group (Figure 3H). Conversely the colonic mucosa from *Rhapontici Radix* extract-treated mice exhibited considerable improvement as evidenced by diminished dysplastic changes and partial restoration towards normal morphological characteristics in certain mucosal regions. When compared to the control group myeloperoxidase (MPO) levels in the colonic mucosa of model mice were significantly elevated and correspondingly the inflammation scores for *Rhapontici Radix* extract-treated mice were substantially lower than those recorded for the model group (Figure 3G).

In addition, the results of the ELISA showed that the level of pro-inflammatory cytokines in the colon tissue of model mice was significantly increased, including TNF-α,IL-1β and IL-6, and anti-inflammatory cytokin. The level of IL-10 decreased significantly. Taking *Rhapontici Radix* extract can effectively reverse these changes, significantly reduce the concentrations of TNF-α, IL-6 and IL-1β, and increase the level of IL-10 (Figure 3I–L). These findings show that the extract of *Rhapontici Radix* has the potential to significantly improve the pathological characteristics of colorectal epithelial damage and relieve intestinal inflammation in model mice.

### 3.4. Effects of Rhapontici Radix Extract on Intestinal Microbiota in AOM/DSS-Induced Colorectal Epithelial Neoplasia Mouse Model

**Figure 4:**
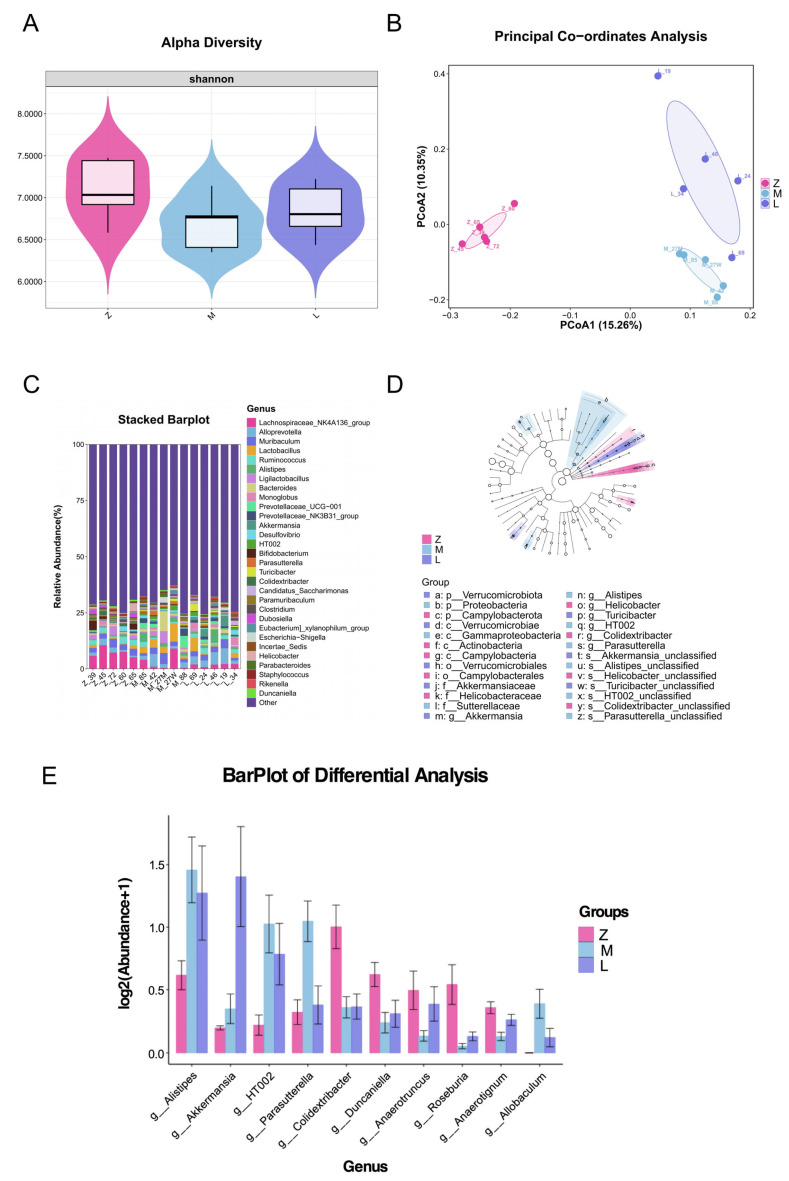
Effects of *Rhapontici Radix* extract on the gut microbiota of the colonic intraepithelial neoplasia mouse model. (**A**) Alpha diversity results of gut microbiota in colonic intraepithelial neoplasia mice after intervention with *Rhapontici Radix* extract. (**B**) Principal component analysis (PCA) of gut microbiota in colonic intraepithelial neoplasia mice after intervention with *Rhapontici Radix* extract. (**C**) Differences in the top 30 abundant bacterial genera in the gut microbiota of colonic intraepithelial neoplasia mice after intervention with *Rhapontici Radix* extract. (**D**) LEfSe analysis of gut microbiota in colonic intraepithelial neoplasia mice post-intervention with *Rhapontici Radix* extract. (**E**) Differential bacterial genera in the gut microbiota of colonic intraepithelial neoplasia mice after intervention with *Rhapontici Radix* extract.

To explore the intervention effect of *Rhapontici Radix* extract on colorectal epithelial neoplasia, we analyzed the intestinal microbiota of normal mice, AOM/DSS-induced colorectal epithelial neoplasia model mice, mesalazine intervention group, and *Rhapontici Radix* extract intervention group. Alpha diversity analysis showed that, compared to normal mice, the model group exhibited significantly reduced species richness and evenness, while the *Rhapontici Radix* extract intervention group showed significant increases in both species richness and evenness (Figure 4A).

At the genus level, analysis of the 30 genera with the most significant differences revealed that *Rhapontici Radix* extract intervention significantly modulated the intestinal microbiota structure of colorectal epithelial neoplasia mice (Figure 4C,D). Specifically, *Rhapontici Radix* extract effectively inhibited the overgrowth of the *Escherichia-shigella* genus and markedly promoted the restoration and colonization of *Akkermansia*, *Bifidobacterium*, and *Lactobacillus* (Figure 4E).

### 3.5. Rhapontici Radix Extract Inhibits the Proliferation of Colonic Organoids from Epithelial Neoplasia Mouse Models

**Figure 5:**
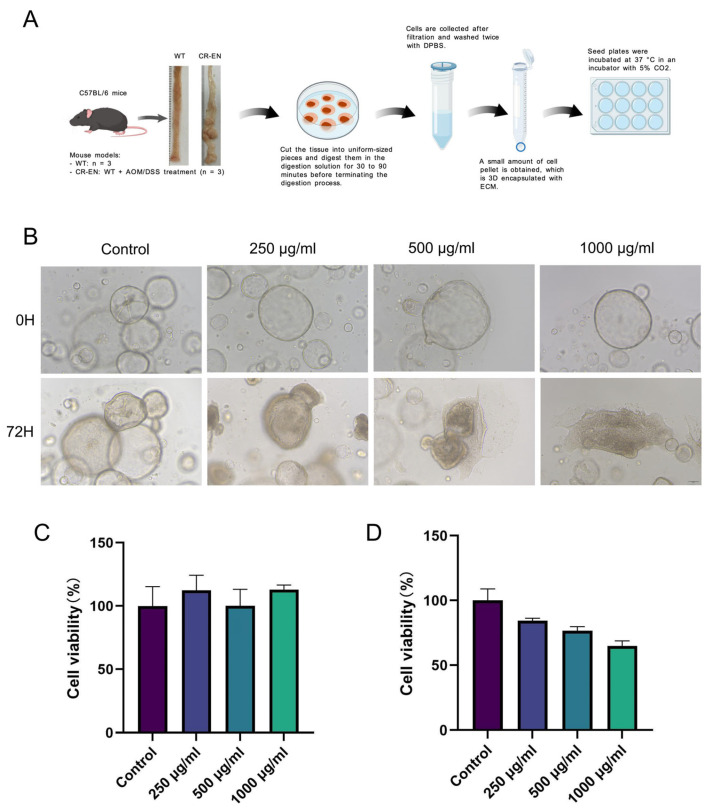
The extract of *Rhapontici Radix* inhibits organoid proliferation in colonic intraepithelial neoplasia mice. (**A**) Extraction methods for organoids from CR-EN mice, and normal mouse colon organoid models. Created with BioGDP.com. (**B**) Optical microscope images of colon organoids from CR-EN mice after intervention with different concentrations of *Rhapontici Radix* extract for 72 h (Original magnification: ×100). (**C**) ATP assay measuring cell viability in colon organoids from normal mice after treatment with varying concentrations of *Rhapontici Radix* extract. (**D**) ATP assay measuring cell viability in colon organoids from colonic intraepithelial neoplasia mice after intervention with different concentrations of *Rhapontici Radix* extract.

We further evaluated the therapeutic effect of *Rhapontici Radix* extract using CAC mouse models (Figure 5A) derived from colorectal cancer patients. Cell viability was assessed by measuring ATP levels, an important energy molecule in live cells that reflects metabolic activity [16]. ATP detection results showed that different concentrations of *Rhapontici Radix* extract had no significant effect on the viability of normal mouse colonic organoids, but it significantly inhibited the viability of organoids derived from epithelial neoplasia mouse models in a dose-dependent manner (Figure 5B–D).

**Figure 6:**
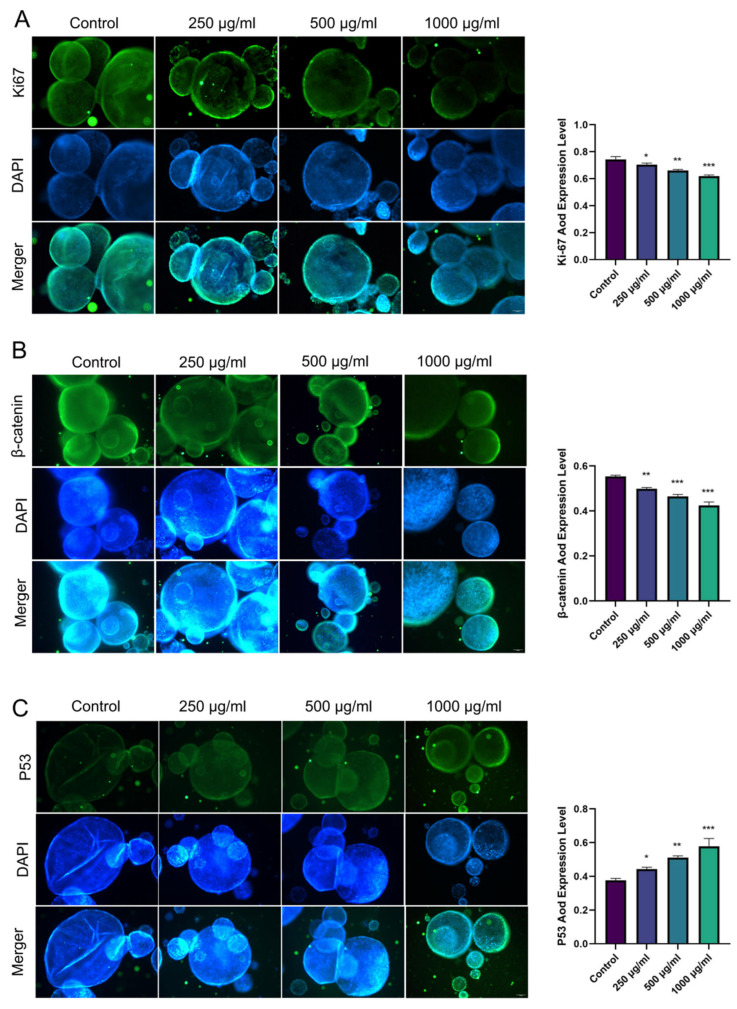
(**A**–**C**) The findings indicate the expression levels of Ki67, β-catenin, and p53 proteins in organoids derived from a colonic intraepithelial neoplasia model in mice, following treatment with various concentrations of *Rhapontici Radix* extract (Original magnification: ×100). Statistical significance was established with * *p* < 0.05, ** *p* < 0.01, and *** *p* < 0.001 when compared to the control group.

Immunofluorescence results showed that after treatment with *Rhapontici Radix* extract, the expression of Ki67 and β-catenin in organoids derived from epithelial neoplasia mouse models significantly decreased, while the expression level of p53 significantly increased (Figure 6A–C). These results indicate that *Rhapontici Radix* extract reduces the proliferative capacity of intestinal epithelial cells in organoids.

### 3.6. Rhapontici Radix Extract Inhibits the Invasion and Migration of HCT116 Colorectal Cancer Cells

**Figure 7:**
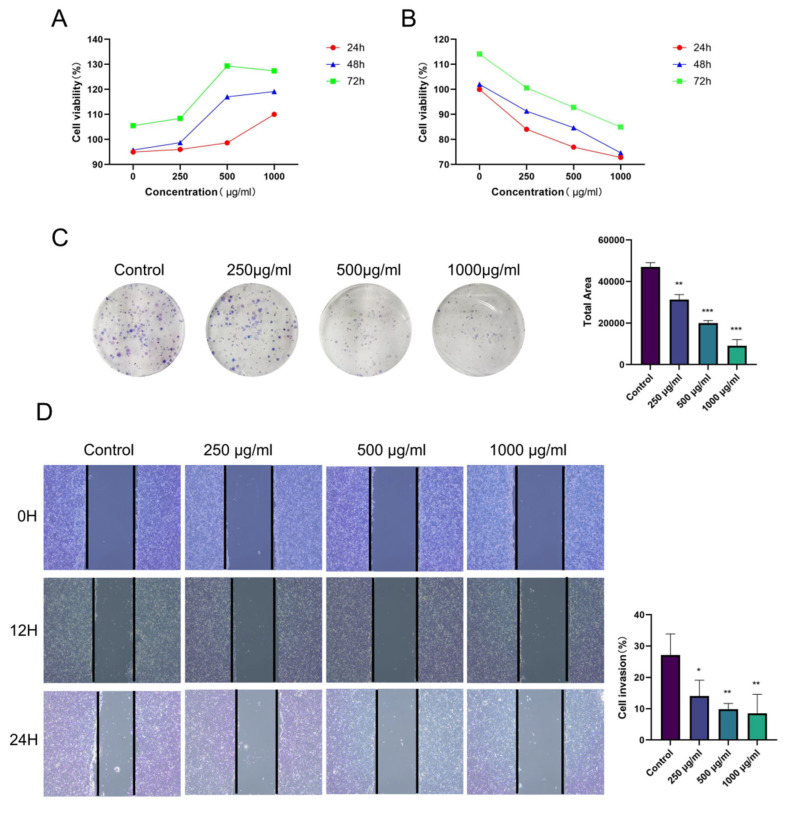
*Rhapontici Radix* extracts demonstrated inhibitory effects on the proliferation of HCT116 colon cancer cells. To further investigate, (**A**) we conducted an MTT assay to evaluate how various concentrations of the extract affected the viability of HCoEpiC cells, while (**B**) the same assay assessed its effects on HCT116 cell viability. (**C**) We also examined the influence of a range of extract concentrations on colony formation in HCT116 cells, and (**D**) evaluated their effects on HCT116 cell migration (Original magnification: ×40). Statistical significance levels, compared to the control group, are indicated as * *p* < 0.05, ** *p* < 0.01, *** *p* < 0.001.

MTT assay results showed that different concentrations of *Rhapontici Radix* extract were applied to HCoEpic cells, a normal human colonic epithelial cell line, to determine the safe dosage range, which was 250, 500, and 1000 μg/mL (Figure 7A). Based on this, a series of concentrations of *Rhapontici Radix* extract were applied to HCT116 cells, a human colorectal cancer cell line, resulting in a significant inhibition of their proliferation (Figure 7B). Moreover, colony formation assays and cell migration assays confirmed that *Rhapontici Radix* extract could significantly reduce the colony formation and migration capabilities of HCT116 cells in a dose-dependent manner (Figure 7C,D).

### 3.7. Rhapontici Radix Extract Inhibits YAP/AKT/PI3K Signaling in Epithelial Neoplasia in C57BL/6 Mice

**Figure 8:**
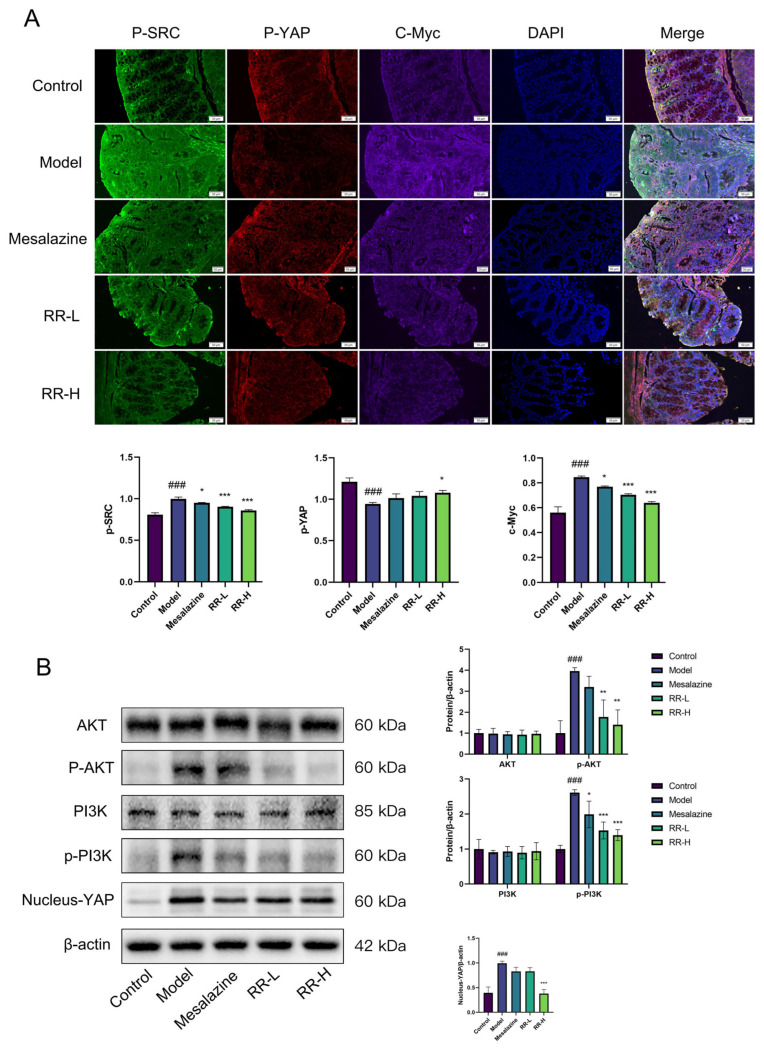
The extract derived from *Rhapontici Radix* influences the modulation of the YAP/AKT/PI3K signaling pathway. (**A**) Immunofluorescence (IF) staining illustrates the levels of p-SRC, p-YAP, and c-MYC proteins within the colons of mice exhibiting colonic intraepithelial neoplasia across various treatment cohorts. (**B**) The findings from Western Blot analysis demonstrate the expression levels of Nucleus-YAP, AKT, p-AKT, PI3K and p-PI3K proteins in the colons of colonic intraepithelial neoplasia mice subjected to different treatment regimens. Statistical significance is indicated as * *p* < 0.05, ** *p* < 0.01, and *** *p* < 0.001 when compared to the model group.Statistical significance was determined with ### *p* < 0.001 when compared to the control group.

Moreover, the results of immunofluorescence assays demonstrated that following specific treatment with *Rhapontici Radix* extract, the expression levels of p-SRC and c-Myc proteins in mouse colon tissues were significantly decreased, while expression level of p-YAP was elevated. (Figure 8A). Western blot analysis further revealed that the protein expression levels of AKT and PI3K in colon tissues from the *Rhapontici Radix* extract-treated group were comparable to those in the model group, but the protein expression levels of Nucleus-YAP, p-AKT, and p-PI3K were lower than those in the model group (Figure 8B). These results illustrate that *Rhapontici Radix* extract can inhibit the carcinogenic process by regulating YAP, AKT, and PI3K-related signaling pathways, thereby exerting a potential anti-colorectal cancer effect.

### 3.8. The Extract of Rhapontici Radix Suppresses the Proliferation of Colorectal Cancer Organoids Through the YAP/AKT/PI3K Signaling Pathway

**Figure 9:**
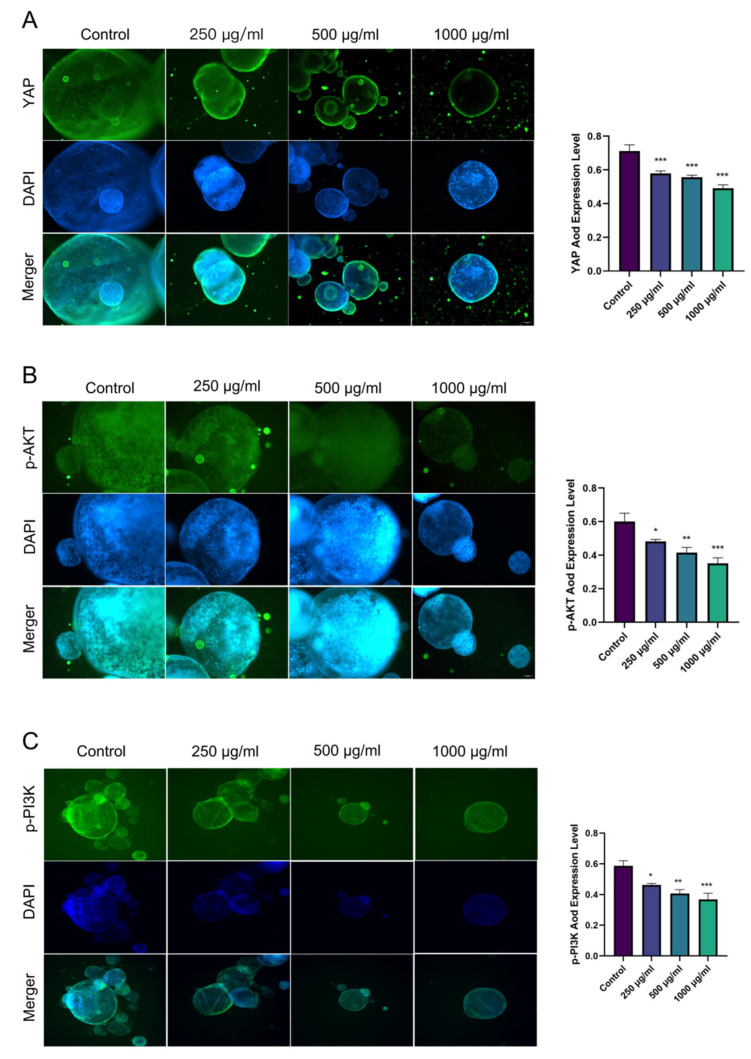
(**A**–**C**) IF results show the expression of YAP, p-AKT, and p-PI3K proteins in organoids from colonic intraepithelial neoplasia model mice after intervention with different concentrations of *Rhapontici Radix* extract (Original magnification: ×100). * *p* < 0.05, ** *p* < 0.01, *** *p* < 0.001 compared to the control group.

Immunofluorescence analyses indicated that treatment with the *Rhapontici Radix* extract resulted in a marked reduction in the levels of YAP, p-AKT, and p-PI3K in organoids derived from mice exhibiting epithelial neoplasia (Figure 9A–C).

## 4. Discussion

Colitis-associated colorectal cancer (CAC) is the most serious complication of inflammatory bowel disease (IBD), and its pathogenesis involves a cascade of chronic inflammation, epithelial dysplasia, and carcinogenesis [1,17]. Compared to sporadic colorectal cancer, CAC presents more aggressive clinical features, including multifocal growth, diagnosis at a late stage, and strong resistance to conventional chemotherapy. Currently, there is a lack of safe and effective chemopreventive drugs for CAC [18,19,20]. Although mesalazine shows some preventive potential, CAC often occurs in IBD patients with long-term chronic inflammation. This makes the study of drugs that can prevent precancerous lesions and inhibit the progression of these lesions a major challenge in CAC research [21,22,23].

In colorectal cancer, YAP and the PI3K/AKT signaling pathway form a critical positive feedback regulatory network that together drives tumorigenesis, progression, and development [24]. Abnormal activation of YAP increases the levels of various receptors and signaling molecules [25]. This elevation enhances the signal output of the PI3K/AKT pathway. Conversely, activated AKT facilitates the nuclear translocation of YAP and enhances its transcriptional activity by inhibiting core Hippo pathway kinases, such as LATS1 and LATS2, which are key regulators of YAP. This self-reinforcing regulatory loop significantly promotes proliferation, survival, maintenance of stemness, and chemoresistance in colorectal cancer cells [25,26]. Therefore, targeting the YAP–PI3K/AKT signaling axis represents a promising therapeutic strategy for more precise treatment of colorectal cancer [27].

To elucidate its key molecular pathway, we focused on the YAP and PI3K/AKT signaling pathways, which play core roles in cell proliferation, survival, and CRC occurrence [28,29,30,31,32,33]. Our bioinformatics analysis suggested that YAP1, AKT1, AKT2, PIK3R2 and PIK3R3 are overexpressed in COAD tissues, consistent with previous studies that consider them oncogenic drivers. The results of multiple immunofluorescence staining showed that the protein expression levels of p-SRC and c-MYC in colorectal cancer tissues were significantly higher than those in adjacent tissues, while the expression level of p-YAP was significantly lower than that in adjacent tissues. This indicates that oncogenic signaling pathways, such as YAP and PI3K/AKT, are activated in colorectal cancer and may exhibit cross-talk.

Preliminary HPLC-MS/MS analysis showed that *Rhapontici Radix* extract contains various natural constituents, including alkaloids, terpenoids, flavonoids, organic acids, and thiophenes. Based on further structural identification and literature cross-validation, several of these components have been reported to exhibit significant antitumor and anti-inflammatory activities. These putative candidate active components and their precise biological effects will be further verified using authentic standards in future studies. For instance, Cyclopamine, a steroidal alkaloid, acts as a specific inhibitor of the Hedgehog signaling pathway, thereby suppressing tumor stem cell self-renewal and tumorigenesis [34]. Parthenolide, a sesquiterpene lactone, induces apoptosis and selectively eliminates cancer stem cells by inhibiting the NF-κB signaling pathway [35]. Embelin, a benzoquinone compound, functions as a small-molecule inhibitor of XIAP (X-linked inhibitor of apoptosis protein), exerting its antitumor effects through the activation of apoptotic pathways [36].Based on the above analysis, it can be speculated that the group of components with definite anti-tumor activity in *Rhapontici Radix* extract is most likely the main pharmacodynamic material basis for its intervention effect on inflammation-cancer transformation during colorectal cancer occurrence.

In a model of colorectal epithelial neoplasia induced by AOM/DSS, our research demonstrated that the extract of *Rhapontici Radix* significantly augmented the primary symptoms associated with this condition. The extract not only alleviated the reduction in colon length observed in the experimental mice, minimized the increase in colon weight, and inhibited tumor progression, but histopathological assessments further indicated its potential to restore colonic mucosal integrity while reducing glandular disorganization and dysplastic changes. The morphological improvements observed are closely associated with its pronounced anti-inflammatory properties. The extract from *Rhapontici Radix* significantly reduced the levels of overexpressed pro-inflammatory cytokines (TNF-α, IL-1β, IL-6) in the colon tissues of the experimental models, while simultaneously elevating the concentration of the anti-inflammatory cytokine IL-10. Furthermore, a significant decrease in myeloperoxidase (MPO) activity and inflammation scores was noted [37,38,39]. Collectively, these findings convincingly demonstrate that the *Rhapontici Radix* extract can effectively mitigate inflammatory injury in the colon by restructuring the inflammatory microenvironment, thus potentially delaying or obstructing the progression from inflammation to cancer.

Moreover, our study found that *Rhapontici Radix* extract has a significant regulatory effect on the intestinal microbiota—an important component of the tumor microenvironment [40]. Model mice exhibited typical dysbiosis, while Rhapontici intervention effectively restored the α diversity and community structure of the microbiota, bringing it closer to a healthy state. Specifically, it promoted the proliferation of barrier-protective bacteria *Akkermansia* and well-known probiotics *Bifidobacterium* and *Lactobacillus*, while inhibiting the growth of genotoxic pathogens such as *Escherichia-shigella* [41,42,43]. Therefore, the improvement of colorectal epithelial neoplasia by Rhapontici may be achieved by reshaping the intestinal microbiota balance.

To validate these findings in a physiologically relevant in vivo-like system and assess the direct anti-proliferative effects of the extract, we introduced colon organoid models. In the colorectal cancer transformation process, abnormal β-catenin activation initiates the transformation process, while p53 dysfunction promotes malignant transformation [44,45,46,47,48]. Additionally, high expression of Ki67, a proliferation marker, reflects uncontrolled proliferation. These three factors are interlinked to drive tumor occurrence and development. Experimental results showed that *Rhapontici Radix* extract dose-dependently inhibited the growth of epithelial neoplasia organoids while having minimal effects on normal organoids. At the molecular level, this growth inhibition was accompanied by downregulation of the proliferation marker Ki67 and the oncogenic protein β-catenin, and upregulation of the tumor suppressor protein p53.

Moreover, our studies in the human colorectal cancer cell line HCT116 further expanded the anti-cancer potential of *Rhapontici Radix* extract. Experiments confirmed that, within a defined safe concentration range determined by preliminary toxicity assays, *Rhapontici Radix* extract effectively inhibited the proliferation, clone formation, and migration capabilities of HCT116 cells in a dose-dependent manner. These findings support its application in inhibiting tumor progression and metastasis.

In line with this, we found that treatment with *Rhapontici Radix* extract significantly reduced the protein levels of Nucleus-YAP and its downstream oncogenic target c-Myc in the colon tissues of CR-EN mice, while increasing the expression of its inhibitory phosphorylated form (p-YAP). Meanwhile, *Rhapontici Radix* extract inhibited the activation of the PI3K/AKT pathway. This finding suggests that *Rhapontici Radix* extract likely targets an upstream regulatory node of the pathway or directly affects PI3K/AKT pathway kinase activity, rather than simply reducing protein synthesis. Crucially, we reconfirmed the inhibitory effects of *Rhapontici Radix* extract on YAP, phosphorylated AKT (p-AKT), and phosphorylated PI3K (p-PI3K) in the organoid models.

Finally, in summary, this study systematically reveals the multi-faceted protective effects of *Rhapontici Radix* extract in colorectal epithelial neoplasia and carcinogenesis. The primary mechanism of the extract may involve the inhibition of the key YAP and PI3K/AKT signaling pathways, thereby blocking abnormal proliferation of colonic epithelial cells, alleviating deterioration of the inflammatory microenvironment, and suppressing malignant behaviors such as cancer cell invasion and metastasis.

These results provide a foundation for further exploring the role of Rhapontici Radix extract in colorectal cancer, but also elucidates its molecular hubs under the multi-component, multi-target action paradigm, thereby creating new research opportunities for traditional Chinese medicine in preventing and treating inflammation-related tumors.

Nevertheless, this study has several limitations. First, only the crude extract of Rhapontici Radix was investigated, and the key active components such as cyclopamine were not isolated or verified individually, so the precise pharmacodynamic basis remains to be clarified. Second, the regulatory effects on YAP and PI3K/AKT signaling pathways were merely demonstrated by expression detection, without rescue or inhibition experiments to confirm the causal relationship, resulting in insufficient mechanistic evidence. Finally, pharmacokinetic characteristics and long-term safety profiles were not evaluated, which limits the support for further clinical application.

## 5. Conclusions

This study systematically elucidates the multi-target action mechanisms of the traditional Chinese medicine *Rhapontici Radix* in the prevention and treatment of colitis-associated colorectal cancer (CAC). The main research conclusions are as follows: In the AOM/DSS-induced mouse CAC inflammation–cancer transformation model, the extract of *Rhapontici Radix* significantly improves intestinal inflammation and pathological tissue damage, effectively reduces tumor burden, and slows the progression of colorectal epithelial neoplasia. Its core mechanism of action may involve the regulation of YAP and PI3K/AKT signaling pathways.

## Data Availability

The original contributions presented in this study are included in the article/Supplementary Material. Further inquiries can be directed to the corresponding author.

## Supplementary Materials

The following supporting information can be downloaded at: https://www.mdpi.com/article/10.3390/biomedicines14050956/s1, HPLC-MS-MS.
